# Enhancing group cognitive‐behavioral therapy for hoarding disorder with between‐session Internet‐based clinician support: A feasibility study

**DOI:** 10.1002/jclp.22589

**Published:** 2018-02-07

**Authors:** Volen Z. Ivanov, Jesper Enander, David Mataix‐Cols, Eva Serlachius, Kristoffer N.T. Månsson, Gerhard Andersson, Oskar Flygare, David Tolin, Christian Rück

**Affiliations:** ^1^ Department of Clinical Neuroscience, Centre for Psychiatry Research Karolinska Institutet Stockholm Sweden; ^2^ Stockholm Health Care Services Stockholm County Council Stockholm Sweden; ^3^ Department of Psychology Stockholm University Stockholm Sweden; ^4^ Department of Behavioural Sciences and Learning Linköping University Linköping Sweden; ^5^ The Institute of Living Yale University School of Medicine New Haven CT USA

**Keywords:** behavioral group therapy, cognitive‐behavioral therapy, hoarding, internet‐based interventions

## Abstract

**Objective:**

Hoarding disorder (HD) is difficult to treat. In an effort to increase efficacy and engagement in cognitive‐behavioral therapy (CBT), we developed and evaluated a novel intervention comprising group CBT combined with between‐session Internet‐based clinician support for people with HD.

**Method:**

Twenty participants with HD received group CBT combined with an Internet‐support system enabling therapist–participant communication between group sessions.

**Results:**

The treatment was associated with a significant reduction on the Saving Inventory—Revised (SI‐R) and a large effect size (Cohen's *d *= 1.57) was found at posttreatment. Treatment gains were maintained at the 3‐month follow‐up. Group attendance was high and no participants dropped out from treatment prematurely. Between‐session motivational support from the therapist was most frequently mentioned as the main strength of the system.

**Conclusion:**

The results of this study support adding Internet‐based clinician support to group CBT for HD to increase treatment adherence and, potentially, improve the overall efficacy of CBT.

## 1 INTRODUCTION

Hoarding disorder (HD) is characterized by an inability to discard one's possessions, resulting in obstructive and hazardous degrees of clutter throughout the sufferer's home (American Psychiatric Association, 2013). Epidemiological studies suggest a point prevalence of at least 1.5% for HD (Nordsletten et al., 2013). Individuals with HD experience a high degree of functional (Nordsletten, Fernandez de la Cruz, Billotti, & Mataix‐Cols, 2013) and work‐related impairment (Tolin, Frost, Steketee, Gray, & Fitch, 2008). The condition can also pose significant health and safety risks for caregivers (Drury, Ajmi, Fernandez de la Cruz, Nordsletten, & Mataix‐Cols, 2014) and the surrounding community (Frost, Steketee, & Williams, 2000).

Before the inclusion of HD in the *Diagnostic and Statistical Manual of Mental Disorders* (DSM–5), hoarding was considered a subtype of obsessive‐compulsive disorder (OCD) and treated accordingly, mainly with exposure and response prevention (ERP) (Mataix‐Cols, Marks, Greist, Kobak, & Baer, 2002) or with selective serotonin reuptake inhibitors (SSRIs) (Mataix‐Cols, Rauch, Manzo, Jenike, & Baer, 1999). However, studies have consistently found that OCD patients who have hoarding symptoms (some of whom may have included individuals with HD) are approximately 50% less likely to respond to ERP, SSRIs, or a combination of the two (Bloch et al., 2014). OCD patients with hoarding symptoms are also more likely to drop out from ERP (Mataix‐Cols et al., 2002). The poor compliance and modest outcomes associated with traditoinal ERP in individuals with hoarding symptoms have led to the development of specialized cognitive‐behavioral therapy (CBT) protocols for HD (Steketee & Frost, 2006). These multimodal protocols typically include motivational interviewing; exposure to nonacquiring; training in sorting, discarding, and organizing; and cognitive restructuring. Specialized CBT for HD has shown promise across several clinical trials, reducing hoarding symptoms by around 25%–30% (e.g., Muroff, Steketee, Bratiotis, & Ross, 2012; Steketee, Frost, Tolin, Rasmussen, & Brown, 2010). According to two recent systematic reviews, specialized CBT for HD, appears to be equally efficacious when delivered individually or in a group format (Thompson, Fernández de la Cruz, Mataix‐Cols, & Onwumere, 2017; Tolin, Frost, Steketee, & Muroff, 2015).

Treatment retention in CBT for HD is far from ideal (attrition rates have ranged from 29% to 33%; Gilliam et al., 2011; Tolin, Frost, & Steketee, 2007) and outcomes are still modest, with the majority of patients (65%) scoring within the clinical range of symptom burden after receiving CBT (Tolin et al., 2015). Moreover, individual CBT for HD is highly time‐consuming, typically including around 30 in‐clinic and home‐based sessions (Steketee et al., 2010), and access to trained therapists specialized in CBT for HD is still very limited. CBT in a group setting might be a more cost‐effective alternative to individual CBT, with the additional benefits of providing social support (Rodriguez et al., 2016) and decreasing stigma (Schmalisch, Bratiotis, & Muroff, 2010). However, the modest treatment efficacy of CBT is similar in both individual and group CBT formats (Gilliam et al., 2011; Muroff et al., 2012).

One frequently proposed barrier to improved treatment outcomes is the low level of treatment engagement, expressed as homework compliance (Tolin et al., 2007) and treatment attrition (Gilliam et al., 2011) among individuals with HD.

Taken together, given the challenges with CBT for HD, new treatment approaches are needed. One approach could be to combine the benefits from different treatment modalities (e.g., cost‐effectiveness and social support in group CBT and flexibility and personalization of treatment in individual CBT). An emerging area in mental health is the use of information technology as an adjunct to conventional face‐to‐face treatments. Typically, such technical advances are websites or mobile applications that serve as support to conventional CBT and facilitate treatment delivery outside the clinic by making homework tasks more accessible, providing memory aids, facilitating self‐monitoring, and providing individualized feedback on progress (Aguilera & Muench, 2012). Recently, an Internet‐support system (COMMIT) was developed with the aim to serve as an adjunct to CBT in a university clinic setting with the goal to improve treatment efficacy and reduce attrition among patients with anxious and depressive disorders (Mansson, Skagius Ruiz, Gervind, Dahlin, & Andersson, 2013). In a pilot study, 15 patients received individual face‐to‐face CBT for 9 weeks and had access to COMMIT between sessions. Individual CBT combined with COMMIT was associated with significant decreases of anxiety and depression symptoms, which were sustained 12 months after treatment. Furthermore, there was no treatment attrition; homework compliance was high; and qualitative interviews with the study participants revealed that COMMIT was perceived as beneficial for both homework compliance and overall treatment engagement. COMMIT combined with CBT has also recently been found to be feasible at an outpatient psychiatric clinic (Mansson, Klintmalm, Nordqvist, & Andersson, 2017). Although preliminary, these findings are promising and suggest that this treatment approach may be a particularly suitable way of cost‐effectively improving efficacy and treatment adherence among individuals with HD.

While Internet‐supported CBT for HD has not yet been studied, one online intervention in a sample of 106 individuals with problematic hoarding has been tested (Muroff, Steketee, Himle, & Frost, 2010). The intervention comprised an online self‐help group, moderated by indviduals with hoarding disorder, and resulted in moderate decreases of the participants’ hoarding symptoms. Despite the modest treatment effects of the intervention, this study provides preliminary support for the feasibility of online interventions for individuals with HD.

The primary aim of this uncontrolled clinical trial was thus to evaluate the feasibility and potential efficacy of a novel treamtent, comprising group CBT combined with between‐session online therapist support for HD. Our hypothesis was that the treatment would result in decreased hoarding symptoms from pretreatment to posttreatment and that these treatment gains would be sustained 3 months after treatment termination. Furthermore, we predicted that the treatment would be acceptable to the participants and that treatment satisfaction and treatment engagement would be high.

## 2 METHODS

### 2.1 Participants

To be included in the study, participants had to be outpatients, 18 years or older, have a primary diagnosis of HD according to DSM‐5 (American Psychiatric Association, 2013), currently be living in Stockholm county, provide written informed consent, have regular access to a computer with Internet and be able to use basic Internet features, have access to a mobile phone, and be able to participate in group sessions at a clinic. Exclusion criteria were current substance dependence or misuse, lifetime diagnoses of bipolar disorder or psychosis, self‐rated depressive symptoms ≥35 on the Montgomery–Åsberg Depression Rating Scale‐Self Report (MADRS‐S;Svanborg & Åsberg, 1994), suicidal ideation (a score of >4 on item 9 in MADRS‐S), psychotropic medication changes within 2 months prior to the treatment. Participants were also carefully asked about other current psychological treatments and were excluded if they reported actively working with their hoarding difficulties in a parallel psychological treatment. Individuals who had ever completed more than 10 sessions of specialized CBT for HD were also excluded.

Demographics and clinical characteristics of the patients are presented in Table 1. Participants were predominantly female (90%), fairly well educated (75% had a college or university degree), and 65% were unemployed or on sick leave. A majority of the participants (55%) reported problems with excessive acquisition and most had good insight into their hoarding difficulties (75%). At inclusion, the most common comorbidities were generalized anxiety disorder (30%) and major depressive disorder (25%). Fifty percent of the participants were using psychotropic medication. Informed written consent was obtained from all the study participants prior to inclusion in the study. The regional ethical board in Stockholm, Sweden approved the study (2015/1476‐31/5) and the trial was registered at clinicaltrials.gov (NCT02584764).

**Table 1:** Demographic and clinical characteristics of the sample (*n* = 20)

Variable	Mean (*n*)	*SD* (%)
Age in years	53.7	8.8
Female	18	90
Employment status		
Employed	6	30
Unemployed or on sick leave	13	65
Retired	1	5
Education		
High school or lower	4	25
University/college	16	75
Source of referral		
Self‐referral	14	70
Clinical referral	6	30
Current use of psychotropic medication	10	50
DSM‐5 specifiers		
Excessive acquisition	11	55
Good insight	15	75
Poor insight	5	25
MADRS‐S total score	18.8	8.0
CGI‐S	4.35	0.75
Current comorbidity		
Generalized anxiety disorder	6	30
Major depressive disorder	4	25
Social anxiety disorder	2	10
Panic disorder	1	5

*Note*. DSM‐5 = *Diagnostic and Statistical Manual of Mental Disorders*, 5th ed.; MADRS‐S = Montgomery–Åsberg Depression Rating Scale—self report; CGI‐S = Clinical Global Impression—Severity.

### 2.2 Recruitment

**Figure 1:**
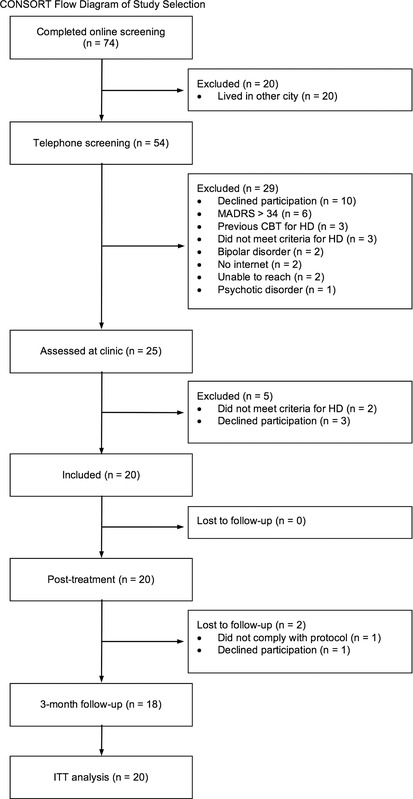
Flowchart of participants throughout the trial *Note*. ITT = intention‐to‐treat; HD = hoarding disorder; MADRS‐S = Montgomery–Åsberg Depression Rating Scale, self‐report version; CBT = cognitive‐behavioral therapy.

Participant flow throughout the study is presented in Figure 1. Patients were recruited through self‐referral or clinical referral at two clinics specialized in treatment of obsessive‐compulsive and related disorders from October 2015 to March 2016. As a first step of the recruitment, 74 individuals underwent an online screening, which comprised the Hoarding Rating Scale‐Self Report (HRS‐SR) (Tolin, Frost, & Steketee, 2010), MADRS‐S, the Alcohol Use Disorders Identification Test (AUDIT) (Saunders, Aasland, Babor, de la Fuente, & Grant, 1993), and the Drug Use Disorders Identification Test (DUDIT) (Berman, Bergman, Palmstierna, & Schlyter, 2005). General information about the participants (demographics, phone numbers, etc.) was also collected at this stage. All individuals who completed the online screening questionnaires, and lived in Stockholm County, were later contacted and assessed via telephone. Participants who clearly did not meet criteria for inclusion were excluded at this stage. The final step of the recruitment comprised an assessment at a psychiatric clinic. Participants meeting inclusion criteria at the clinical assessment were provided with written and verbal information about the study and signed an informed consent form. During the same visit, participants were introduced to the COMMIT system and were also asked to upload photographs of the rooms in their home.

### 2.3 Assessment and outcomes

During the clinical assessment prior to treatment, diagnostic criteria and specifiers for HD were assessed by the first author with the Structured Interview for Hoarding Disorder (SIHD) (Mataix‐Cols, Billotti, Fernandez de la Cruz, & Nordsletten, 2013) and the Mini‐International Neuropsychiatric Interview (MINI) (Sheehan et al., 1998), was used to determine comorbid Axis I diagnoses using DSM‐IV criteria.

All clinician‐rated instruments and self‐report measures were administered at pretreatment, posttreatment and at 3‐month follow‐up, with the exception of measures of treatment satisfaction and participant experiences, which were only administered at posttreatment. Additionally, the Saving Inventory—Revised (SI‐R) was also administered at mid‐treatment (session 8) and the HRS‐SR, along with questions about homework compliance, was administered in COMMIT every week during treatment. At the 3‐month follow‐up, self‐report questionnaires were filled out during a group session at the clinic and clinician‐rated measures were conducted over the phone.

#### 2.3.1 Primary outcome measure

The primary outcome measure was the SI‐R, a self‐administered questionnaire measuring hoarding difficulties divided into three domains (difficulty discarding, clutter, and excessive acquisition;Frost, Steketee, & Grisham, 2004). The questionnaire contains 23 items scored from 0 (*no difficulties*) to 4 (*extreme difficulties*), which are summed to a total score, ranging from 0 to 40.

#### 2.3.2 Secondary outcome measures

Secondary outcome measures comprised self‐report measures including the Saving Cognitions Inventory (SCI; Steketee et al., 2003), HRS‐SR (Tolin et al., 2010), and EuroQol (EQ‐5D; Group, 1990). In addition, we included the clinician‐rated measures Clinical Global Impression (CGI; Guy, 1976) and Global Assessment of Functioning (GAF; Jones, Thornicroft, Coffey, & Dunn, 1995) which were rated by the first author.

Levels of clutter were assessed with Clutter Image Rating (CIR; Frost, Steketee, Tolin, & Renaud, 2008). The CIR is a visual assessment of the clutter dimension of hoarding and comprises nine photographs depicting increasing levels of clutter in a bedroom, kitchen, and living room, rated on a scale from 1 to 9. In order to assess levels of clutter confidently (Fernandez de la Cruz, Nordsletten, Billotti, & Mataix‐Cols, 2013), the study participants were asked to upload photographs of their bedroom, kitchen, and living room in COMMIT. The photographs were independently rated with the CIR by one clinical psychologist and one research assistant who were blind to whether the photographs were taken before or after treatment. For every photograph, the mean score of the two raters was used as a composite score and subsequently, a mean composite score was calculated across all the rooms for every participant.

#### 2.3.3 Treatment activity and adherence

Adherence to group CBT was defined as the average attended group sessions during treatment. We defined treatment dropout as coming to a mutual agreement with the group facilitator to terminate treatment. Compliance with homework was rated by the participants in COMMIT. Throughout every week of treatment, participants were asked to report how many hours they had devoted to their homework assignment, and to what degree they had completed the assignment (scores: 0 = “not at all,” 1 = “in part,” 2 = “completely”). We measured treatment activity in COMMIT by averaging the number of messages sent and received by the participants and the duration of time logged in the system.

#### 2.3.4 Treatment satisfaction and participant experiences

Treatment satisfaction was assessed with Client Satisfaction Questionnaire (CSQ‐8; Nguyen, Attkisson, & Stegner, 1983), which comprises eight items, assessing satisfaction with a specific healthcare or counseling service. The scale ranges from 8 to 32 with higher scores indicating greater satisfaction with a service. Participants’ experiences of using COMMIT were evaluated with a questionnaire, specifically designed for this study, comprised of qualitative questions regarding the strengths and weaknesses of COMMIT. Participants were also asked to rate the level of difficulty of learning how to use COMMIT on a Likert‐type scale ranging from 1 (*very easy to learn*) to 6 (*very difficult to learn*).

### 2.4 Treatment

#### 2.4.1 Group treatment

Participants received a treatment comprising group CBT with added Internet‐based clinician support between group sessions. Group treatment was delivered at two different sites specializing in obsessive‐compulsive and related disorders. Group CBT comprised 16 weekly, 2.5‐hour sessions. Each group treatment was facilitated by two psychologists with several years of experience in treating obsessive‐compulsive and related disorders. The first author was one of the psychologists at one of the clinics and provided weekly supervision and a 1‐day training course for the group facilitators at the other clinic. Group CBT followed a manual based on the current psychological model for HD (Tolin et al., 2017). The manual comprises psychoeducation about CBT and HD, goal‐setting, motivation enhancement, executive skills training, cognitive restructuring, mindfulness‐based skills to accept and tolerate negative emotions, and relapse prevention. Group sessions started with a review of the homework assignment from the previous week, followed by a review and exercises regarding a specific hoarding‐related topic. In‐session practice of discarding was encouraged from the second week of treatment and the latter part of sessions 2–15 was devoted to sorting and discarding personal items. Individual homework assignments were agreed upon at the end of every session.

#### 2.4.2 Internet support, COMMIT

Participants had access to the online support‐system COMMIT (Mansson et al., 2013) between group sessions. COMMIT could be accessed via a personal computer, tablet, or mobile phone and the connection was encrypted with a secured sockets layer (SSL). After inclusion to the treatment, participants received personal identification numbers to access COMMIT. In order to increase security levels, an additional, temporary password was sent via short message service (SMS) every time the participant attempted to access the system.

The content in COMMIT was tailored for individuals with HD and differed slightly from the original version aimed at clients with anxiety and depressive disorders (Mansson et al., 2013). This adaption was made due to the potential deficits in attention and executive functioning that have been reported among individuals with HD (Woody, Kellman‐McFarlane, & Welsted, 2014), which might make it difficult to manage a homework load coupled with extensive and technologically complicated tasks independently. COMMIT included a digital copy of the current treatment manual, including homework assignments and treatment goals, as well as questionnaires for monitoring treatment progression. After every group session, the therapists uploaded the homework assignments to COMMIT. The short‐term and long‐term treatment goals were decided on at the beginning of treatment and could be edited and updated by the participants throughout treatment. The participants were also asked to upload two photographs of the living area they had worked on every week, one prior to and one after, a decluttering session. Photographs could be uploaded to COMMIT securely by the participant, using a smart phone or computer. One additional feature in COMMIT was the possibility to communicate with a group therapist through a built‐in message system. Participants were encouraged to send inquiries about the treatment and report treatment progress or barriers. The therapist's role was to enhance treatment engagement by providing personalized feedback on homework assignments, motivational support, and practical guidance in the decluttering process of the participants’ homes between clinic sessions. Participants could message the therapist at any time and messages from the participants were answered within 36 hours, with the exception of weekends. Two brief questionnaires were also administered weekly in COMMIT: the five‐item hoarding questionnaire HRS‐SR and questions about homework compliance. The participants had access to a visual graph depicting their weekly HRS‐SR ratings throughout the entire treatment.

### 2.5 Statistical analyses

Analyses were by intention‐to‐treat. Missing data were deemed to be missing at random using logistic regression models (*p *= .17–.69). The effects of time on the continuous outcome measures were analyzed with linear mixed effects models (Verbeke, 2009). The models included fixed effects for time, with participant‐varying intercepts included as a random effect in the model. Pairwise comparisons were performed to test if treatment gains were maintained at 3‐month follow‐up. Within‐group effect sizes for change between pretreatment and posttreatment were calculated using Cohen's *d*. The associations between time spent on homework and time using COMMIT, as well as the association between these variables and changes in outcomes from pretreatment to posttreatment were analyzed with Pearson's correlations. All statistical analyses were conducted in STATA 13.1 (StataCorp, College Station) and the significance level was set to *p* < .05.

## 3 RESULTS

### 3.1 Primary outcome measure

Table 2 shows the estimated means and standard errors obtained from mixed‐effects models for the SI‐R at every assessment point. The mean reduction from baseline to mid‐treatment on SI‐R total scores was −7.95, 95% CI [−12.82, −3.08], *p* < .001, with further improvements observed from mid‐treatment to posttreatment of an additional −9.50 points, 95% CI [−14.37, −4.63], *p* < .001. From pretreatment to posttreatment, there was a large within‐group effect size; *d* = 1.57, 95% CI [0.85, 2.27]. The gains made during the treatment were sustained from posttreatment to 3‐month follow‐up, as no significant differences on the SI‐R total scores were observed, mean increase 1.07 points, 95% CI [−3.97, 6.10], *p* = .68. In total, the mean reduction on the SI‐R total scores from pretreatment to follow‐up was of −16.38 points, 95% CI [−21.42, −11.34], *p* < .001, representing a large within‐group effect size; *d *= 1.40, 95% CI [0.68, 2.11].

**Table 2:** Primary and secondary outcome measures at every assessment point: Effect sizes, Cohen's *d*, including 95% confidence intervals between pretreatment and posttreatment

Estimated means (*SE*)					
Measure	Pretreatment	Mid‐treatment	Posttreatment	3‐Month follow‐up	Cohen's *d*, 95% CI
SI‐R total	65.10 (2.38)	57.15 (2.38)	47.65 (2.38)^b^	48.72 (2.47)	1.57 (0.85–2.27)
SI‐R clutter	29.3 (1.25)	27.25 (1.25)	22.85 (1.25)^b^	23.76 (1.28)	1.22 (0.53–1.89)
SI‐R difficulty discarding	20.70 (1.06)	18.95 (1.06)	14.95 (1.06)^b^	15.32 (1.10)	1.17 (0.49–1.84)
SI‐R acquiring	15.10 (0.90)	10.95 (0.90)	9.85 (0.90)^b^	9.61 (0.93)	1.30 (0.61–1.98)
SCI total	103.45 (5.49)	–	79.27 (5.58)^b^	83.99 (5.78)	1.08 (0.40–1.75)
SCI emotional attachment	44.75 (2.82)	–	30.93 (2.87)^b^	33.45 (2.98)	1.26 (0.57–1.95)
SCI control	14.60 (0.94)	–	13.35 (0.95)^b^	14.57 (0.99)	0.34 (−0.30–0.97)
SCI responsibility	23.45 (1.45)	–	18.45 (1.48)^b^	19.28 (1.55)	0.81 (0.15–1.46)
SCI memory	20.65 (1.66)	–	16.42 (1.69)^b^	14.30 (1.66)	0.63 (−0.1–1.27)
CIR^a^	5.24 (0.36)	–	4.00 (0.38)^b^	N/A	0.96 (0.15–1.75)
GAF	52.65 (2.13)	–	62.77 (2.17)^b^	65.95 (2.25)	−1.21 (−1.89–0.52)
EQ‐5D	0.77 (0.02)	–	0.74 (0.02)	0.77 (0.02)	0.31 (−0.33 – 0.95)

*Note*. SI‐R = Saving Inventory—Revised; SCI = Saving Cognitions Inventory; CIR = clutter image rating; GAF = Global Assessment of Functioning; N/A = no available data; EQ‐5D = EuroQol; *SE* = standard error; CI = confidence interval; *p* = *p* value.

a Mean household rating.

b *p* < 0.05, *p* values refer to comparisons between pretreatment and posttreatment.

### 3.2 Secondary outcome measures

As shown in Table 2, overall, we observed significant improvements from pretreatment to posttreatment, and large effect sizes on all secondary outcome measures, with the exception of general health status and quality of life measured with the EQ‐5D. Moreover, all improvements from pretreatment to posttreatment were largely maintained at 3‐month follow‐up.

**Figure 2:**
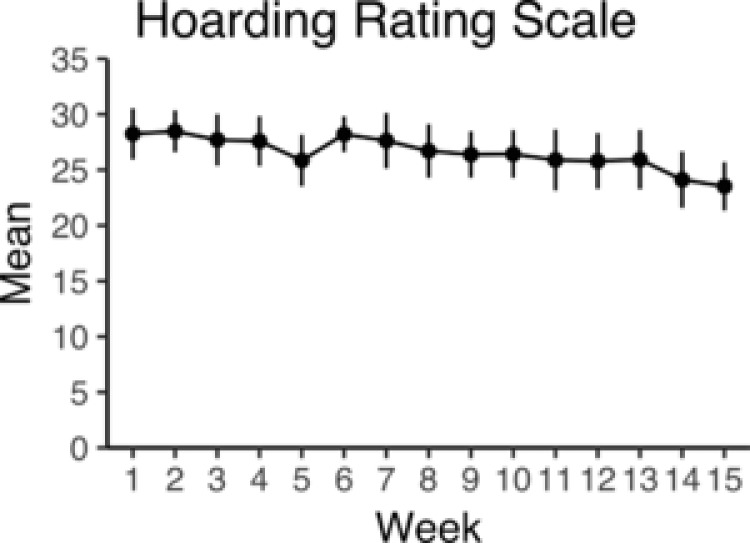
Weekly scores on the self‐administered Hoarding Rating Scale, HRS‐SR (including 95% CIs)

Ten (50%) of the participants uploaded photographs of the same living areas as at pretreatment and could thus be scored with the CIR and included in the analysis. CIR scores improved from baseline to posttreatment with −1.24 points, 95% CI [−1.57, −0.83], *p* < .001. The reliability between the two blind raters was excellent, intraclass correlation = 0.94, 95% CI [0.89, 0.98]. At 3‐month follow‐up, none of the participants uploaded photographs, despite being encouraged by their therapists. Mean HRS‐SR scores decreased significantly, *p *< .05, with −4.11, 95% CI [− 6.46, −1.76] points from Week 1 to Week 15. Weekly mean ratings including 95% CIs on the HRS‐SR are shown in Figure 2.

Nineteen and 17 participants had complete clinical data at posttreatment and 3‐month follow‐up, respectively. Ten participants, 50%; 95% CI: 28%– 0.72%, and 11 participants, 65%; 95% CI: 38%–85%, at posttreatment and follow‐up respectively, were rated as much improved or very much improved on the GCI‐I.

Five participants, 25%; 95% CI [0.10, 0.50] did not fulfill diagnostic criteria for HD when assessed with SIHD at posttreatment and at 3‐month follow‐up.

### 3.3 Treatment activity and adherence

#### 3.4 Group CBT

All participants who were included in the study completed the group treatment and attended an average of 14.3, standard deviation (*SD*) = 1.6, out of 16 group sessions. One participant could not attend five consecutive group sessions due to work related commitments but wished to remain in treatment and continued communicating with the therapist during this period. Another participant was removed from the trial after the group treatment due to difficulties complying with the study protocol and was not included in the 3‐month follow‐up.

#### 3.5 COMMIT

All participants who attended group treatment used COMMIT between group sessions. Each participant accessed COMMIT for 70 minutes on average (range = 11–727, median = 29), sent two, *SD* = 2.3, messages and received three, *SD* = 1.7, messages every week. Therapists spent an average of 18 minutes, *SD* = 9, per patient every week, range = 6–28 minutes, median = 20 minutes, supporting patients in COMMIT.

#### 3.6 Homework

Nineteen (95%) participants provided data on homework activity. These participants reported spending 4 hours, *SD* = 2.7, on homework assignments every week, range = 0.4–9.9 hours, median = 3.2, and rated their average homework compliance as 1.04, *SD* = 0.2, indicating that on average, the participants completed their weekly homework “in part.” There was a significant correlation between time spent on homework and time accessing COMMIT, *r* = 0.46, *p* < .05. However, neither time spent on homework, homework compliance, nor time accessing COMMIT was associated with change on any outcome measures from pretreatment to posttreatment.

#### 3.7 Treatment satisfaction and participant experiences

Seventeen (85%) patients rated their satisfaction and acceptance of treatment by completing the CSQ‐8 at posttreatment. The mean score on the questionnaire was 25.9, *SD* = 5.4, indicating that, on average, patients were very satisfied with the received treatment. Seven (41%) participants reported that they were very pleased; eight (47%) that they were pleased; one (6%) was neither pleased nor displeased; and one (6%) was somewhat displeased with the treatment provided.

At posttreatment, data on the experience of using COMMIT were available for 11 (55%) participants. Overall, COMMIT was perceived as easy to learn, mean = 1.8, *SD* = 0.9. The most commonly mentioned strength was receiving motivation and support from the therapist. Weaknesses that were reported by the participants largely concerned technical aspects of the system, such as difficulties logging into the system, issues with uploading photographs, and the design of the interface. The most common response to this question was, however, that the support system did not have any weaknesses (data available upon request).

## 4 DISCUSSION

One of the biggest challenges in treating HD is to keep patients motivated and engaged throughout the treatment process. In this study, we tested the acceptability and potential efficacy of a novel treatment, combining a group CBT protocol (Tolin, 2017) with between‐session Internet‐based clinician support (Mansson et al., 2013) for adults with HD. The intervention was associated with improvements on the primary outcome and almost all secondary outcomes. Within‐group effect sizes were overall large, *d *= 0.96–1.57, at posttreatment and sustained at 3‐month follow‐up. At posttreatment and follow‐up, 25% of participants no longer met diagnostic criteria for HD according to a structured diagnostic interview. During treatment, group attendance was high and no participants dropped out. Treatment satisfaction was high, and COMMIT was frequently used and perceived as easy to master by the participants. Motivational support from the therapist was most frequently mentioned as the main strength of the system and there was a significant correlation between using the online support and time spent on homework assignments.

Overall, the effect size on the SI‐R, *d *= 1.57, in this trial was somewhat larger compared to a previous trial of group CBT, 16–20 sessions, for HD, *d *= 1.31, which also reported an attrition rate of 33% (Gilliam et al., 2011; Muroff et al., 2009). However, the effect size was slightly smaller compared to group CBT combined with four, *d *= 2.03, or eight, *d *= 3.36, home visits (Muroff et al., 2012). Although comparisons between different trials should be made cautiously, our results raise the question of whether adding COMMIT to CBT might produce similar outcomes, but at a lower cost, compared to CBT combined with home visits. The importance of COMMIT for symptom improvement is, however, in need of further empirical support since we did not find an association between the use of COMMIT and change on any of the outcome measures.

Despite the notable reductions of hoarding symptoms, when taking the secondary outcomes in consideration, the overall effect of the treatment may be considered as modest, also suggesting that there is room for further improvement. As in most trials of CBT for HD, the majority of our participants remained impaired or distressed by their hoarding difficulties after treatment and only 25% were free from a diagnosis of HD at the end of the treatment and follow‐up. Moreover, levels of clutter in the participants’ homes, as measured with the CIR, only decreased by one point following the intensive 16‐week treatment. It is, however, generally considered that greater reductions of clutter might take considerably longer than the length of standard CBT protocols (Tolin et al., 2015).

The lack of treatment attrition in this study is noteworthy, given the high rates of attrition (29–33%) and the commonly fluctuating levels of motivation previously reported among people with HD (Gilliam et al., 2011; Tolin et al., 2007). It is plausible that the Internet support contributed to increased treatment activity and adherence. The positive association between the time spent on homework and the use of COMMIT, as well as the results from the treatment evaulation questions administered at posttreatment, both support the importance of COMMIT for treatment adherence. The majority of participants reported feeling supported and motivated by using COMMIT and experienced that regularly uploading photographs boosted their motivation and facilitated monitoring progress. Naturally, there was some variability in the participants’ satisfaction with and use of COMMIT. Some particpants did not perceive the support‐system as helpful and mainly reported dissatisfaction with several technical aspects of COMMIT. Further improvements of the technical functions are thus warranted prior to future studies including COMMIT.

In terms of feasibility, the treatment was conducted in a clinical psychiatric setting at two different clinics with therapists who were managing a regular workload. The additional time the online support required was not perceived as overwhelming. The average time the therapists spent weekly on every participant (19 minutes) means that a group of six to eight participants, facilitated by two therapists, would require an additional 60–80 minutes of therapist time per week. Although this might be considered as burdensome in many clinical settings, COMMIT is likely to be notably more cost‐effective than adding home visits to group CBT.

### 4.1 Limitations

This study should be interpreted in light of several limitations. First, due to the lack of a control group, it cannot be ruled out that the observed improvements were merely due to the passage of time or the concurrent receipt of additional treatments. However, since HD is highly likely to be a chronic disorder (Tolin, Meunier, Frost, & Steketee, 2010), it is improbable that spontaneous remission would have occurred during the duration of treatment. Furthermore, without a control group, we cannot infer that it was the treatment itself that produced the outcomes or to what extent the addition of online support contributed, as the improvements and high retention may be due to other variables, such as nonspecific factors, including expectancy, social desirability, therapist allegiance, group cohesion, and mutual aid (Schmalisch et al., 2010).

The second limitation pertains to the generalizability of our results. In conformity with all the previous treatment trials for HD, the majority of participants in our study were female, highly educated, and with sufficient insight to seek help for their difficulties (Tolin et al., 2015). Thus, the generalizability of our results to males who generally make smaller improvements in CBT for HD (Tolin et al., 2015), individuals with low education, and individuals with poor insight is questionable. Future treatment studies should aim to resolve this issue by actively recruiting individuals of both genders and with a broader range of educational levels and insight.

Finally, all the clinician ratings of global functioning and improvement were conducted by the first author. Future trials would benefit from independent clinician ratings.

### 4.2 Future directions

One further question to be considered in future studies is what “blend” of face‐to‐face treatment and Internet‐based therapist support would produce optimal treatment outcomes. Although we chose to add online clinician support to group CBT throughout the whole treatment, other combinations are also worth taking into consideration. Given that further treatment gains after completed CBT are rare (Muroff, Steketee, Frost, & Tolin, 2013), future studies may wish to examine whether the addition of therapist support through COMMIT after treatment termination could lead to long‐term improvements and come at a low cost. Alternatively, Internet support could be offered prior to CBT as a stepped care approach for individuals at risk for developing HD or instead of face‐to‐face CBT for highly motivated individuals with HD. Indeed, Internet delivered CBT, as a stand‐alone treatment, has been proven to be very successful for HD‐related disorders such as OCD (Andersson et al., 2012) and body dysmorphic disorder (Enander et al., 2016) and might be effective for a subset of the patients with HD. A third alternative would be to combine CBT and Internet support with other modalities, for instance, contingency management, which has shown promising outcomes when combined with CBT (Worden, Bowe, & Tolin, 2017).

## 5 CONCLUSIONS

Our results suggest that combining group CBT with between‐session Internet‐based clinician support for HD is feasible, acceptable, and associated with high treatment engagement and a significant reduction of hoarding symptoms at posttreatment and 3‐month follow‐up. These preliminary results await confirmation in future controlled trials.
